# Supplementary material for: Influence of Growth Medium Composition on Physiological Responses of Escherichia coli to the Action of Chloramphenicol and Ciprofloxacin
Source: BioTech (Basel). 2023 Jun 1;12(2):43. doi: 10.3390/biotech12020043 (PMC10296315; doi:10.3390/biotech12020043)
Supplement: Supplementary file 1 [file biotech-12-00043-s001.zip › Figure S2-new.pdf]

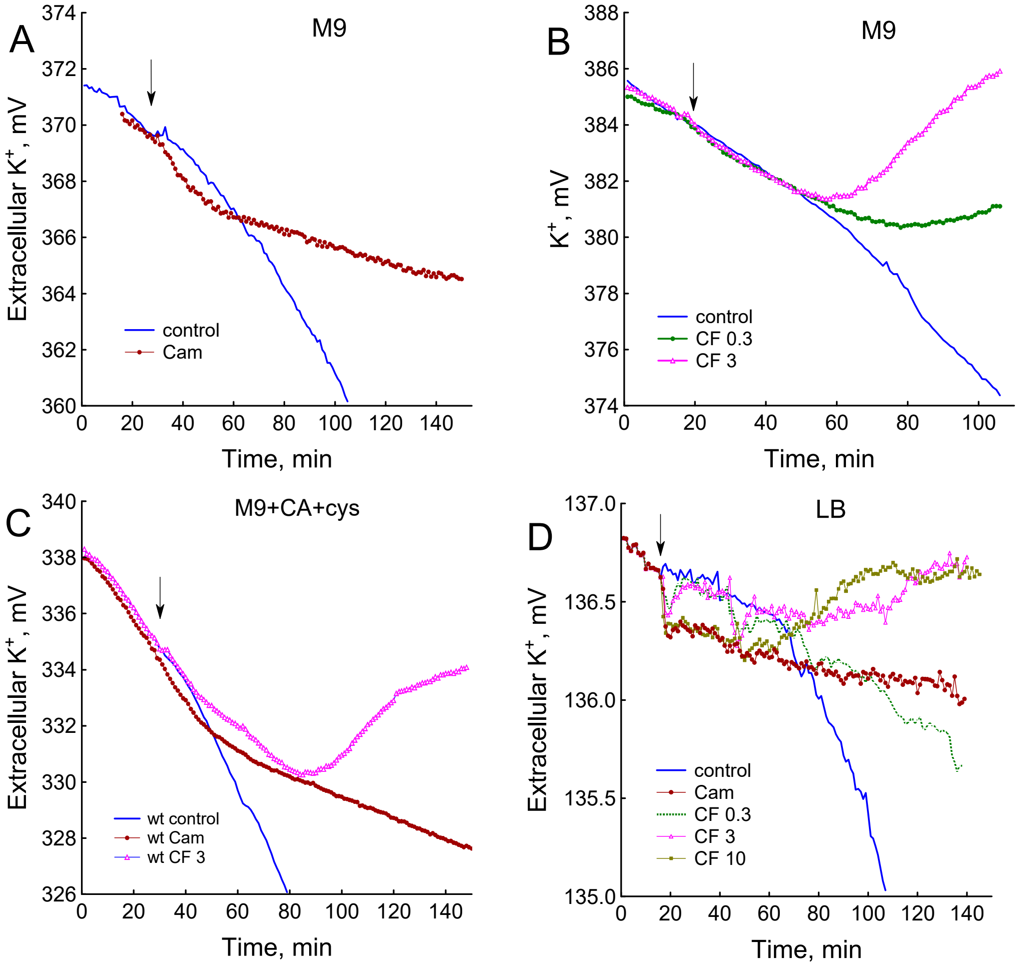

Figure S2. Effect of chloramphenicol (**A**) and ciprofloxacin (**B**) on K^+ uptake by *E. coli* BW25113 cells during growth in M9 medium (**A, B**), in M9+CA+cys medium (**C**) and in LB medium (**D**). 25 μg/ml chloramphenicol or 0.3 and 3 μg/ml ciprofloxacin were added at the time indicated by the arrow.
