# Supplementary material for: Influence of Growth Medium Composition on Physiological Responses of Escherichia coli to the Action of Chloramphenicol and Ciprofloxacin
Source: BioTech (Basel). 2023 Jun 1;12(2):43. doi: 10.3390/biotech12020043 (PMC10296315; doi:10.3390/biotech12020043)
Supplement: Supplementary file 1 [file biotech-12-00043-s001.zip › Figure S3-new.pdf]

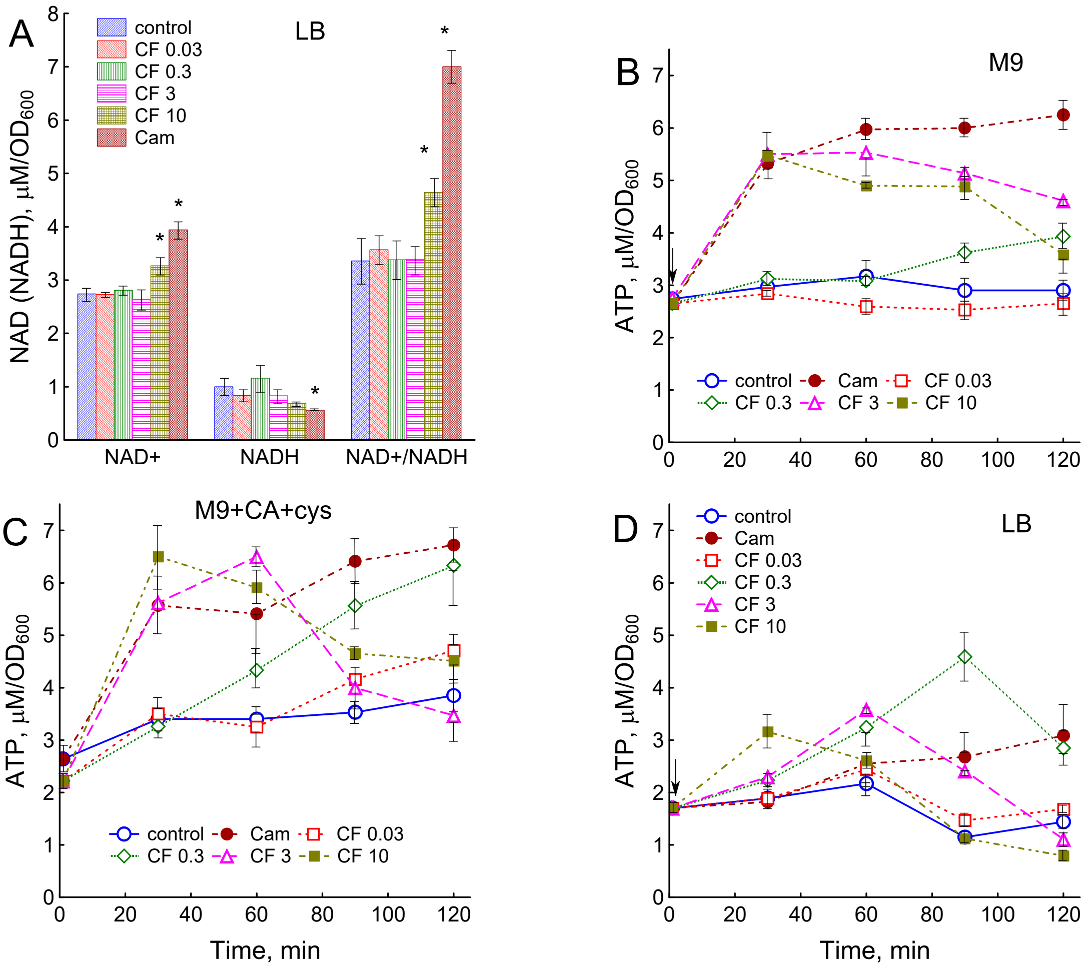


**Figure S3**. The effect of chloramphenicol and ciprofloxacin on the NAD/NADH ratio and ATP level during *E. coli* growth in different media. (**A**) NAD/NADH in LB medium. (**B**) ATP in M9 medium. (**C**) ATP in M9+CA+cys medium. (**D**) ATP in LB medium.
