# Supplementary material for: Influence of Growth Medium Composition on Physiological Responses of Escherichia coli to the Action of Chloramphenicol and Ciprofloxacin
Source: BioTech (Basel). 2023 Jun 1;12(2):43. doi: 10.3390/biotech12020043 (PMC10296315; doi:10.3390/biotech12020043)
Supplement: Supplementary file 1 [file biotech-12-00043-s001.zip › Figure S1-new.pdf]

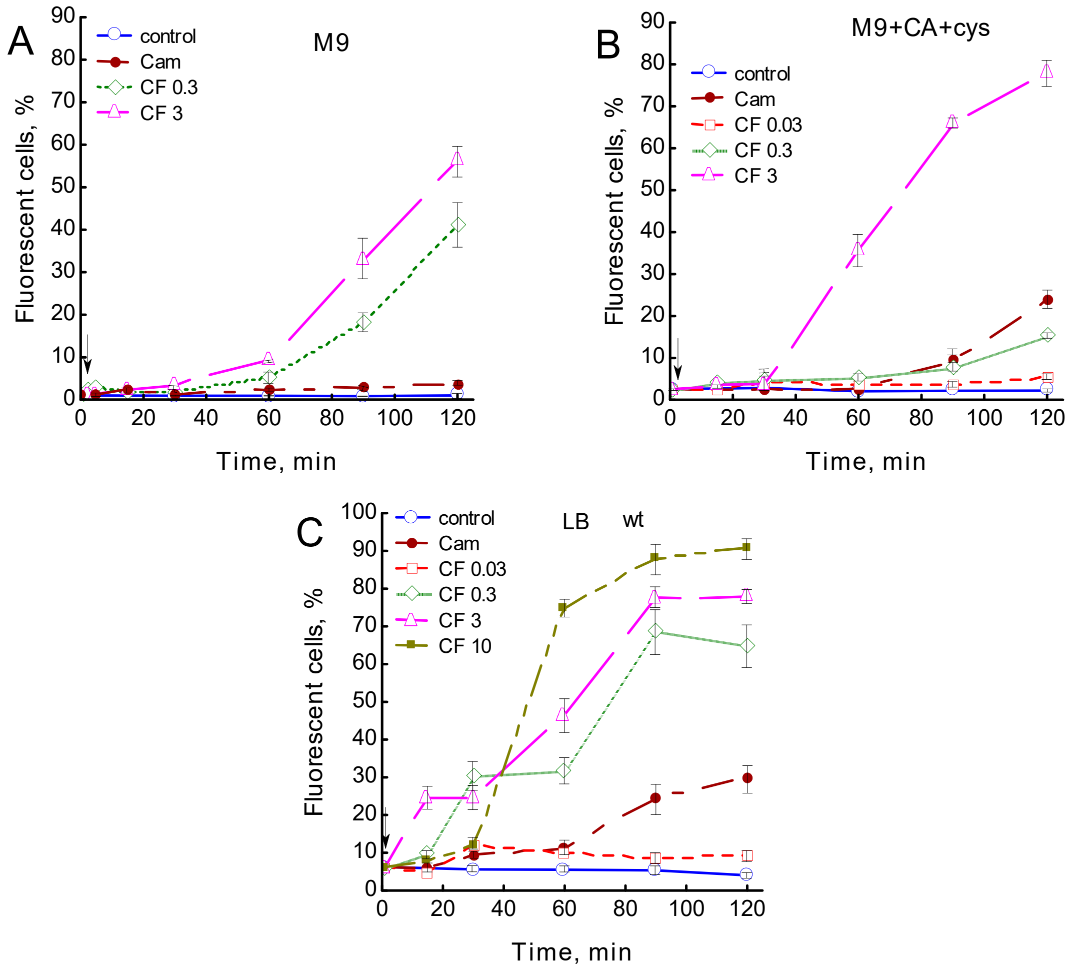

Figure S1. Changes in the number of depolarized cells (stained with fluorescent dye DiBAC) during exposure to antibiotics in different media. (**A**) minimal M9 medium; (**B**) M9 supplemented with casamino acids and cystine (M9+CA+cys); (**C**) LB medium. Chloramphenicol (Cam, 25 μg/ml) and ciprofloxacin (CF, 0.03, 0.3, 3 and 10 μg/ml) were added at the point indicated by the arrow.
